# A self-harm series and its relationship with childhood adversity among adolescents in mainland China: a cross-sectional study

**DOI:** 10.1186/s12888-018-1607-0

**Published:** 2018-02-01

**Authors:** Azhu Han, Gengfu Wang, Geng Xu, Puyu Su

**Affiliations:** 1 Department of Maternal, Child and Adolescent Health, School of Public Health, Anhui Medical University, No.81 Meishan Road, Hefei, Anhui 230032 China; 2 Department of Maternal, Child and Adolescent Health, School of Public Health, Tianjin Medical University, No. 22 Qixiangtai Road, Tianjin, 300070 China; 3 Anhui Provincial Key Laboratory of Population Health and Aristogenics, Hefei, Anhui China

**Keywords:** Self-harm, Childhood adversity, Childhood maltreatment, Childhood peer victimization, Childhood family life stress events, Adolescents

## Abstract

**Background:**

Self-harm (SH) is an emerging problem among Chinese adolescents. The present study aimed to measure the prevalence of SH behaviours and to explore the relationship between childhood adversity and different SH subtypes among Chinese adolescents.

**Methods:**

A total of 5726 middle school students were randomly selected in three cities of Anhui province, China, using a stratified cluster sampling method. SH was categorized into five subtypes (highly lethal self-harm, less lethal self-harm with visible tissue damage, self-harm without visible tissue damage, self-harmful behaviours with latency damage and psychological self-harm). Multivariate logistic regression was used to explore the relationships between childhood adversity and different subtypes of adolescent SH.

**Results:**

The prevalence rates of highly lethal self-harm, less lethal self-harm with visible tissue damage, self-harm without visible tissue damage, self-harmful behaviours with latency damage and psychological self-harm were 6.1, 20.4, 32.0, 20.0 and 23.0%, respectively. Childhood sexual abuse and physical peer victimization were associated with each SH subtype with adjusted odds ratios (AORs) ranging from 1.23 to 1.76. Highly lethal self-harm was associated with childhood physical peer victimization, sexual abuse, emotional abuse, and emotional neglect. The less lethal SH subtypes (i.e., less lethal self-harm with visible tissue damage, self-harm without visible tissue damage, self-harmful behaviours with latency damage and psychological self-harm) were associated with childhood peer victimization, family life stress event scores and childhood sexual abuse.

**Conclusions:**

A high prevalence of SH exists among Chinese adolescents. The association of childhood adversity with SH merits serious attention in both future research and preventive interventions.

**Electronic supplementary material:**

The online version of this article (10.1186/s12888-018-1607-0) contains supplementary material, which is available to authorized users.

## Background

### The prevalence of SH

Self-harm (SH) is characterized by a wide range of behaviours and intentions, including attempted hanging, impulsive self-poisoning, and superficial cutting [1], which aim to relieve a terrible state of mind [2] or communicate stress [3]. SH behaviour is a significant public health issue worldwide with a high prevalence. Swannell et al. [4] estimated that the lifetime prevalence of non-suicidal self-injury (NSSI) among community adolescents was 17.2% worldwide and ranged from 1.5% to 54.8% across different areas. A meta-analysis of adolescents indicated that the prevalence rates of NSSI in the eastern, central and western regions of mainland China were 21.9, 23.0, and 2.1%, respectively [5]. Salient differences in the prevalence of SH exist among different areas, even within a country. Two key factors may explain the varied reporting rates of SH in different studies. The first is that scholars have not reached a consensus on the definition of SH, and the second is that different studies have used different methods to measure SH.

Performing an international comparison of the prevalence of SH among adolescents is difficult. Some researchers have argued that the specific method of self-injury should be considered first rather than the intent behind the self-injurious behaviours when studying these behaviours among adolescents [1]. To the best of our knowledge, the intention underlying SH is very complex, even for the same form of SH. In contrast, investigations of the specific type of SH are easier and more precise. Generally, SH is classified into two categories based on whether the participant has suicidal intentions: suicidal SH (e.g., suicide [6, 7]) and non-suicidal SH (e.g., NSSI [8, 9]). The International Classification of Diseases 10th Revision (ICD-10) defined intentional self-harm as purposely self-inflicted poisoning or injury, including suicide (attempted). The concept of intentional self-harm is mainly focused on apparent self-harmful behaviours. The definition of NSSI in the Diagnostic and Statistical Manual of Mental Disorders, 5th Edition (DSM-5) was intentional self-inflicted injury performed with the expectation of physical harm without suicidal intent [10]. However, both the ICD-10 and DSM-5 classified SH as the result of obvious damage to an individual’s own self, whereas implicit forms of self-harmful behaviours were neglected. Based on previous studies [1, 11], five subtypes of SH were proposed according to the severity of the negative impact of SH: highly lethal self-harm, less lethal self-harm with visible tissue damage, self-harm without visible tissue damage, self-harmful behaviours with latency damage, and psychological self-harm. It is hoped that this classification of SH will lead to a better understanding of SH in China.

### The relationship between childhood adversity and SH among adolescents

Childhood adversity refers to difficult and unpleasant situations and experiences in childhood, including physical, sexual, or emotional abuse, neglect and poverty [12]. Approximately half of adolescents have experienced at least one type of childhood adversity [8, 13]. Emerging evidence supports a link between childhood adversity and SH. Exposure to childhood abuse increases the risk for later depression [14], NSSI [8] and suicidal behaviours [6, 7], including a significant dose relationship between childhood maltreatment and lifetime suicide attempts [6]. Moreover, a history of childhood sexual abuse increases the likelihood of attempted suicide by almost 4-fold in childhood (4–12 years of age) compared to adulthood (20–29 years of age) [7]. Another study indicates that childhood maltreatment by parents and others increase the risk of NSSI in adolescents, with a cumulative effect with additional perpetrators [8].

Being bullied during childhood not only increases internalizing/externalizing problems and antisocial behaviours [15] but is also associated with SH in adolescence [9]. In a previous study, frequent bullying reported by three informant groups (children, parents, and teachers) predicted subsequent psychiatric disorders, and the number of childhood peer victimizations reported by teachers served as the strongest predictor [16]. In addition, younger children who were victimized reported significantly more NSSI than older children did [17].

Stressful life events, including family financial difficulties, family conflicts, parental death and divorce [13, 18–21], were more likely to cause SH among adolescents. Previous studies have reported that negative life event experiences or stressful life event scores and their prevalence are significantly higher in individuals who perform deliberate self-harm (DSH) or attempted suicide [22–24]. Furthermore, a significant dose-response relationship has been found for the number of negative life events and suicidal behaviours [24].

To date, few studies have detailed the association between comprehensive childhood adversity and SH among Chinese adolescents. Information on the prevalence of each subtype of SH among Chinese adolescents is also lacking. Therefore, the main aims of the present study were to 1) investigate the prevalence of an SH series in mainland China and 2) further explore possible associations between childhood adversity (i.e., childhood maltreatment, childhood peer victimization, and childhood family life stress events) and the five SH subtypes.

## Methods

### Study design and participants

This study was part of the research project “Adolescent Health and Risky Behaviours in Anhui Province”. A 3-stage, random, cluster sampling approach was employed to select participants in Anhui province in the middle of China. Three cities (Tongling, Chuzhou, and Fuyang) were randomly selected in the first stage. Tongling, Chuzhou, and Fuyang are located in southern, middle and northern Anhui, respectively. In the second stage, one regular middle school and one regular high school were randomly selected from each city. In the third stage, eight target classrooms within each school were randomly selected.

In the selected classes, a total of 6032 students were invited to take part in this study. Of these students, 205 refused to participate in the study and 67 were absent from school at the time of the survey. Thus, the questionnaire was completed by 5760 (95.5%) students. After screening to determine the completeness of the questionnaires, we obtained an effective sample of 5726 students, including 2848 males (49.7%) and 2878 females (50.3%). The students were aged 12 to 18 years, and the mean age was 14.81 years.

### Procedure

All of the students in the selected classes were invited to participate voluntarily in this study. Students who were absent from class were excluded from the study. Trained interviewers administered anonymous questionnaires in the absence of teachers and to avoid any potential information bias. Each student sat at a separate table. All data were collected in December 2013.

### Ethical approval

The study received approval from the Biomedicine Ethical Committee of Anhui Medical University. All of the participants were fully informed about the purpose of this investigation and were invited to participate voluntarily. Additionally, written informed consent was obtained from the targeted school, each participating student and one of the student’s parents.

### Measurements

#### Childhood adversity

In this study, three main types of childhood adversity (i.e., childhood maltreatment, childhood peer victimization, and childhood family life stress events) were investigated.

##### Childhood maltreatment

The questions assessing childhood physical abuse (PA), emotional abuse (EA), sexual abuse (SA), physical neglect (PN), and emotional neglect (EN) were based on the Childhood Trauma Questionnaire Short Form (CTQ-SF) [25]. Questions regarding childhood experiences were rated on a 5-point scale with response options ranging from never to very often (1 = never, 2 = rarely, 3 = sometimes, 4 = often, and 5 = very often). The score for each type of maltreatment ranged from 5 to 25. The Chinese version of the questionnaire showed acceptable reliability (internal consistency reliability coefficients ranging from 0.78 to 0.90 and test-retest reliability ranging from 0.79 to 0.88). The CTQ-SF cut-off scores used in this study were as follows: PA ≥ 8; EA ≥ 9; SA ≥ 6; PN ≥ 8; and EN ≥ 10 [26].

##### Childhood peer victimization

The measures of childhood peer victimization were based on previous studies [27, 28], and two new items were created based on the Chinese social context. The response options were based on a five-point scale as follows: 1 = never; 2 = rarely; 3 = sometimes; 4 = often; and 5 = very often. First, a standard definition of victimization (qifu) in China was given [29]. Then, the participants were asked how often they had been bullied by peers during their childhood.

Three types of childhood peer victimization were measured. Physical peer victimization was measured by two items: (a1) hitting, kicking, pushing, shoving, or locking indoors (new item); and (a2) blackmailing for money or damaging things. Verbal peer victimization was measured by two items: (b1) calling mean names or making fun or teasing in a hurtful way and (b2) saying mean things about an accent (new item). Relational peer victimization was measured by two items: (c1) excluding others from their group of friends or leaving others out of things on purpose and (c2) telling lies or spreading false rumours about others or sending mean notes and trying to make others unpopular. In this study, we used the criterion that students were bullied at least 2 to 3 times per month to evaluate occurrences of physical, verbal and relational victimization [27].

##### Childhood family life stress events

The following ten items assessing childhood family life stress events were based on previous studies in China [30, 31]: (1) family financial difficulties; (2) parents often fight or quarrel; (3) family trauma (e.g., earthquake, fire, and theft); (4) parents overconsume alcohol or are addicted to gambling; (5) disabled family member; (6) parental absence; (7) family member involved in a crime; (8) parental divorce; (9) death of family member; and (10) family member with a serious illness. The response options for each item were 0 = no and 1 = yes. Aggregated scores for each participant were calculated and divided into three groups: 0 score group (*n* = 2049); 1–2 score group (*n* = 2337); and 3–10 score group (*n* = 1340).

##### Self-harm

In this study, we considered SH as a series of self-inflicted and intentional behaviours that caused physical and psychological harm. Based on previous studies [1, 11], we developed 39 items: nine items for highly lethal self-harm; eight items for less lethal self-harm with visible tissue damage; eight items for self-harm without visible tissue damage; nine items for self-harmful behaviours with latency damage; and five items for psychological self-harm. The candidate behaviours for highly lethal self-harm were traditional forms of suicide, which could be similar to suicide attempts in our study. Types of less lethal self-harm with visible tissue damage and self-harm without visible tissue damage were consistent with current reports for non-suicidal SH, including NSSI and DSH; these are actions with a low likelihood of death. Additionally, two new types of SH, including self-harmful behaviours with latency damage and psychological self-harm, were assessed in our study. Although these two types of SH do not result in tissue damage, they may be the primary SH category in adolescents and are usually ignored by researchers.

Participants were asked whether they had self-harmed in the past 6 months. The response option was dichotomized as follows: 0 = no and 1 = yes. Highly lethal self-harm was measured by nine items: (1) hanging; (2) jumping from a high place; (3) poisoning (e.g., herbicide, pesticide, or carbon monoxide); (4) cutting blood vessels deliberately (e.g., cutting wrist, neck, or fatal parts of the blood vessels); (5) stabbing; (6) electrocution; (7) drowning; (8) overdosing (e.g., hypnagogues); and (9) recreational drug ingestion as SH. Less lethal self-harm with visible tissue damage was measured by eight items: (1) cutting (e.g., arms, legs, or other parts except for fatal parts of the body); (2) burning; (3) self-biting; (4) scratching; (5) gouging; (6) carving words or symbols into skin; (7) sticking needles or pins into skin; and (8) interfering with wound healing. Self-harm without visible tissue damage was measured by eight items: (1) self-hitting; (2) banging head or fist against something; (3) deliberate frostbite; (4) pinching; (5) malaxating; (6) binding; (7) pulling hair; and (8) choking. Self-harmful behaviours with latency damage were measured by nine items: (1) exercising to hurt oneself; (2) denying oneself a necessity as punishment; (3) stopping medication or starving with intent to cause harm; (4) deliberate recklessness (e.g., risk-taking with cars or trains); (5) having intercourse with another (not for the purpose of money or love); (6) overconsuming alcohol (e.g., alcoholism or drinking beyond one’s endurance capacity); (7) smoking too much; (8) overeating; and (9) staying up too late (not for working, learning, or entertainment). Psychological self-harm was measured by five items: (1) closing oneself off (forcing oneself to reduce or cease contact with the outside word); (2) distancing oneself from friends on purpose; (3) making oneself unpopular among friends on purpose; (4) insulting oneself; and (5) despising oneself. Each subtype of SH was dichotomized as one or more items versus none.

##### Covariates

We controlled for the potential influence of several sociodemographic variables and depression on SH, including gender (female or male), age (calculated from date of birth and survey date), relationship with mother (good or poor), relationship with father (good or poor), family structure (nuclear family, large family, single-parent family, or other), self-perceived family status (bad, general, or good) and only child (yes or no). Many studies have found that a consistently cited risk factor for SH is depression [32–34], which is also known to induce SH [1, 35]. Zung’s Self-Rating Depression Scale (SDS) [36] was adopted in this study to evaluate depression. The SDS consists of 20 items, with a total number of points ranging from 20 to 80. The questionnaire has been revised in China [37]. A previous study recommended the criterion of an SDS total score greater than or equal to 40 to indicate that participants were at risk for depression [37].

### Reliability and validity of the measurements

The questionnaire was assessed by three experts in this field, and some items were revised based on their suggestions. Then, the questionnaire was retested (1-week interval) with 156 senior and junior students to guarantee that the content and language were suitable for the study population. The Kappa values ranged from 0.82 to 0.95. Moreover, the consistency of the test was examined, with a range from 0.72 to 0.84.

### Statistical analysis

The analyses were performed using SPSS for Windows (version 19.0; SPSS Inc., Chicago, IL, USA). Descriptive statistics were reported for all factors and the prevalence of each SH subtype. Univariate logistic regression analysis was performed to explore the relationships between covariates (e.g., the sociodemographic variables and depression), childhood adversity and each SH subtype. Multivariate logistic regression models were performed to evaluate the relationships between childhood adversity and each SH subtype, with adjustment for sociodemographic variables and depression. All significance tests in this study were evaluated using two-sided tests with a significance level of 0.05.

## Results

### The prevalence of each SH subtype

In this sample, the prevalence rates of highly lethal self-harm, less lethal self-harm with visible tissue damage, self-harm without visible tissue damage, self-harmful behaviours with latency damage and psychological self-harm were 6.1%, 20.4%, 32.0%, 20.0% and 23.0%, respectively. Each SH subtype was more common among female students (see Table 1). Additionally, the prevalence of non-involvement in SH and the frequency of students who were involved overlapped between each SH subtype, as shown in Additional file 1: Table S1 and Additional file 2: Table S2.

**Table 1:** Prevalence of self-harm by sample characteristics (*N* = 5726)

Variables	n (%)	Highly lethal self-harm	Less lethal self-harm with visible tissue damage	Self-harm without visible tissue damage	Self-harmful behaviors with latency damage	Psychological self-harm
		n (%)	n (%)	n (%)	n (%)	n (%)
Gender						
Female	2878 (50.3)	197 (6.8)	763 (26.5)	972 (33.8)	600 (20.8)	749 (26.0)
Male	2848 (49.7)	152 (5.3)	405 (14.2)	862 (30.3)	545 (19.1)	567 (19.9)
Age						
12 years	876 (15.3)	28 (3.2)	133 (15.2)	217 (24.8)	91 (10.4)	119 (13.6)
13 years	919 (16.0)	59 (6.4)	191 (20.8)	297 (32.3)	170 (18.5)	200 (21.8)
14 years	842 (14.7)	56 (6.7)	180 (21.4)	239 (28.4)	156 (18.5)	165 (19.6)
15 years	803 (14.0)	56 (7.0)	174 (21.7)	234 (29.1)	170 (21.2)	185 (23.0)
16 years	892 (15.6)	63 (7.1)	178 (20.0)	302 (33.9)	187 (21.0)	234 (26.2)
17 years	996 (17.4)	62 (6.2)	226 (22.7)	382 (38.4)	267 (26.8)	295 (29.6)
18 years	398 (7.0)	25 (6.3)	86 (21.6)	163 (41.0)	104 (26.1)	118 (29.6)
Self-perceived family status						
Bad	800 (14.0)	57 (7.1)	183 (22.9)	304 (38.0)	187 (23.4)	227 (28.4)
General	4377 (76.4)	256 (5.8)	877 (20.0)	1370 (31.3)	846 (19.3)	967 (22.1)
Good	549 (9.6)	36 (6.6)	108 (19.7)	160 (29.1)	112 (20.4)	122 (22.2)
Relationship with mother						
Good	4373 (76.4)	227 (5.2)	843 (19.3)	1358 (31.1)	795 (18.2)	927 (21.2)
Poor	1353 (23.6)	122 (9.0)	325 (24.0)	476 (35.2)	350 (25.9)	389 (28.8)
Relationship with father						
Good	3815 (66.6)	191 (5.0)	700 (18.3)	1135 (29.8)	666 (17.5)	779 (20.4)
Poor	1911 (33.4)	158 (8.3)	468 (24.5)	699 (36.6)	479 (25.1)	537 (28.1)
Only child						
Yes	2469 (43.1)	113 (4.6)	399 (16.2)	711 (28.8)	435 (17.6)	481 (19.5)
No	3257 (56.9)	236 (7.2)	769 (23.6)	1123 (34.5)	710 (21.8)	835 (25.6)
Family structure						
Nuclear family	3792 (66.2)	225 (5.9)	765 (20.2)	1190 (31.4)	761 (20.1)	867 (22.9)
Lager family	1353 (23.6)	91 (6.7)	276 (20.4)	447 (33.0)	256 (18.9)	310 (22.9)
Single-parent family	469 (8.2)	26 (5.5)	99 (21.1)	152 (32.4)	99 (21.1)	112 (23.9)
Other	112 (2.0)	7 (6.3)	28 (25.0)	45 (40.2)	29 (25.9)	27 (24.1)
Childhood physical peer victimization						
Yes	4494 (78.5)	213 (4.7)	778 (17.3)	1264 (28.1)	761 (16.9)	879 (19.6)
No	1232 (21.5)	136 (11.0)	390 (31.7)	570 (46.3)	384 (31.2)	437 (35.5)
Childhood verbal peer victimization						
Yes	3325 (58.1)	152 (4.6)	525 (15.8)	832 (25.0)	522 (15.7)	552 (16.6)
No	2401 (41.9)	197 (8.2)	643 (26.8)	1002 (41.7)	623 (25.9)	764 (31.8)
Childhood relational peer victimization						
Yes	4143 (72.4)	175 (4.2)	615 (14.8)	1039 (25.1)	578 (14.0)	674 (16.3)
No	1583 (27.6)	174 (11.0)	553 (34.9)	795 (50.2)	567 (35.8)	642 (40.6)
Physical abuse						
Yes	425 (7.4)	71 (16.7)	155 (36.5)	236 (55.5)	171 (40.2)	205 (48.2)
No	5301 (92.6)	278 (5.2)	1013 (19.1)	1598 (30.1)	974 (18.4)	1111 (21.0)
Emotional abuse						
Yes	632 (11.0)	112 (17.7)	263 (41.6)	356 (56.3)	257 (40.7)	308 (48.7)
No	5094 (89.0)	237 (4.7)	905 (17.8)	1478 (29.0)	888 (17.4)	1008 (19.8)
Sexual abuse						
Yes	499 (8.7)	88 (17.6)	206 (41.3)	268 (53.7)	210 (42.1)	220 (44.1)
No	5227 (91.3)	261 (5.0)	962 (18.4)	1566 (30.0)	935 (17.9)	1096 (21.0)
Physical neglect						
Yes	379 (6.6)	77 (20.3)	166 (43.8)	229 (60.4)	179 (47.2)	198 (52.2)
No	5347 (93.4)	272 (5.1)	1002 (18.7)	1605 (30.0)	966 (18.1)	1118 (20.9)
Emotional neglect						
Yes	228 (4.0)	63 (27.6)	111 (48.7)	144 (63.2)	116 (50.9)	124 (54.4)
No	5498 (96.0)	286 (5.2)	1057 (19.2)	1690 (30.7)	1029 (18.7)	1192 (21.7)
Family life stress event scores						
0	2049 (35.8)	95 (4.6)	239 (11.7)	417 (20.4)	258 (12.6)	255 (12.4)
1–2	2337 (40.8)	144 (6.2)	514 (22.0)	805 (34.4)	484 (20.7)	588 (25.2)
3–10	1340 (23.4)	110 (8.2)	415 (31.0)	612 (45.7)	403 (30.1)	473 (35.3)
Depression						
Yes	3711 (64.8)	301 (8.1)	891 (24.0)	1341 (36.1)	896 (24.1)	1023 (27.6)
No	2015 (35.2)	48 (2.4)	277 (13.7)	493 (24.5)	249 (12.4)	293 (14.5)

### Univariate logistic regression analyses

As shown in Table 2, the univariate analyses revealed a significant association between (1) childhood maltreatment (OR range from 2.43 to 6.96), (2) childhood peer victimization (OR range from 1.87 to 3.51), (3) family life stress events scores (OR range from 1.35 to 3.84) and (4) relationship with both parents (OR range from 0.55 to 0.76) and each SH subtype.

**Table 2:** Unadjusted OR (95% CI) for self-harm by univariate analysis

		Highly lethal self-harm	Less lethal self-harm with visible tissue damage	Self-harm without visible tissue damage	Self-harmful behaviors with latency damage	Psychological self-harm
Gender	Female: Male	1.30 (1.05, 1.62)*	2.18 (1.90, 2.49)***	1.18 (1.05, 1.31)**	1.11 (0.98, 1.27)	1.42 (1.25, 1.60)***
Age		1.07 (1.02, 1.34)*	1.06 (1.02, 1.09)***	1.11 (1.08, 1.14)***	1.17 (1.13, 1.21)***	1.16 (1.12, 1.20)***
Self-perceived family status	Ref: Bad					
	General	0.81 (0.60, 1.09)	0.85 (0.71, 1.01)	0.74 (0.64, 0.87)***	0.79 (0.66, 0.94)**	0.72 (0.60, 0.85)***
	Good	0.92 (0.59, 1.41)	0.83 (0.63, 1.08)	0.67 (0.53, 0.85)***	0.84 (0.65, 1.09)	0.72 (0.56, 0.93)*
Relationship with mother	Good: Poor	0.55 (0.44, 0.70)***	0.76 (0.65, 0.87)***	0.83 (0.73, 0.94)**	0.64 (0.55, 0.74)***	0.67 (0.58, 0.77)***
Relationship with father	Good: Poor	0.59 (0.47, 0.73)***	0.69 (0.61, 0.79)***	0.73 (0.65, 0.83)***	0.63 (0.55, 0.72)***	0.66 (0.58, 0.75)***
Only child	Yes: No	0.61 (0.49, 0.77)***	0.62 (0.55, 0.71)***	0.77 (0.69, 0.86)***	0.77 (0.67, 0.87)***	0.70 (0.62, 0.80)***
Family structure	Ref: Nuclear Family					
	Lager family	1.14 (0.89, 1.47)	1.01 (0.87, 1.18)	1.08 (0.95, 1.23)	0.93 (0.79, 1.09)	1.01 (0.87, 1.16)
	Single-parent Family	0.93 (0.61, 1.41)	1.06 (0.84, 1.34)	1.05 (0.85, 1.29)	1.07 (0.84, 1.35)	1.06 (0.85, 1.33)
	Other	1.06 (0.49, 2.30)	1.32 (0.85, 2.04)	1.47 (0.99, 2.16)	1.40 (0.91, 2.14)	1.07 (0.70, 1.66)
Childhood physical peer victimization	Yes: No	2.49 (1.99, 3.12)***	2.21 (1.92, 2.55)***	2.20 (1.93, 2.50)***	2.22 (1.92, 2.56)***	2.26 (1.97, 2.60)***
Childhood verbal peer victimization	Yes: No	1.87 (1.50, 2.32)***	1.95 (1.71, 2.22)***	2.15 (1.92, 2.40)***	1.88 (1.65, 2.14)***	2.35 (2.07, 2.66)***
Childhood relational peer victimization	Yes: No	2.80 (2.25, 3.48)***	3.08 (2.69, 3.52)***	3.01 (2.67, 3.40)***	3.44 (3.01, 3.94)***	3,51 (3.08, 4.00)***
Physical abuse	Yes: No	3.62 (2.73, 4.81)***	2.43 (1.97, 3.00)***	2.89 (2.37, 3.54)***	2.99 (2.43, 3.68)***	3.51 (2.87, 4.30)***
Emotional abuse	Yes: No	4.41 (3.46, 5.62)***	3.30 (2.77, 3.93)***	3.16 (2.67, 3.74)***	3.25 (2.73, 3.87)***	3.85 (3.25, 4.57)***
Sexual abuse	Yes: No	4.07 (3.14, 5.29)***	3.12 (2.57, 3.78)***	2.71 (2.25, 3.27)***	3.34 (2.76, 4.04)***	2.97 (2.46, 3.59)***
Physical neglect	Yes: No	4.76 (3.60, 6.28)***	3.38 (2.73, 4.19)***	3.56 (2.87, 4.41)***	4.06 (3.28, 5.02)***	4.14 (3.35, 5.12)***
Emotional neglect	Yes: No	6.96 (5.08, 9.52)***	3.99 (3.05, 5.21)***	3.86 (2.93, 5.09)***	4.50 (3.44, 5.88)***	4.31 (3.29, 5.63)***
Family life stress event scores	Ref: 0					
	1–2	1.35 (1.04, 1.76)*	2.14 (1.81, 2.52)***	2.06 (1.79, 2.36)***	1.81 (1.54, 2.14)***	2.37 (2.01, 2.78)***
	3–10	1.84 (1.39, 2.44)***	3.40 (2.84, 4.06)***	3.29 (2.83, 3.83)***	2.99 (2.51, 3.56)***	3.84 (3.23, 4.56)***
Depression	Yes: No	3.62 (2.65, 4.93)***	1.98 (1.71, 2.30)***	1.75 (1.55, 1.97)***	2.26 (1.94, 2.63)***	2.24 (1.94, 2.58)***

Note: 95%CI = 95% confidence interval. * *P* < 0.05; ** *P* < 0.01; *** *P* < 0.001

### The association between each type of childhood adversity and each SH subtype

After adjustment for covariates (e.g., sociodemographic variables that were significant in the univariate analysis and depression scale scores), the associations between childhood adversity and SH in adolescents were weakened. Childhood sexual abuse and physical peer victimization were associated with each SH subtype, with adjusted odds ratios (AORs) ranging from 1.23 to 1.76. Highly lethal self-harm was associated with childhood physical peer victimization, emotional abuse, sexual abuse, and emotional neglect. Childhood peer victimization, family life stress event scores and childhood sexual abuse were associated with less lethal subtypes of SH. Additionally, the associations between childhood adversity and the four less lethal SH subtypes were similar in this study (see Table 3). We also performed a multivariate logistic regression analysis to confirm the relationships between childhood adversity and each SH subtype, with adjustments for all sociodemographic variables and depression. The results showed similar relationships (see Additional file 3: Table S3).

**Table 3:** Multivariable logistic regression analysis showing the AOR (95% CI) between childhood adversity and five subtypes of self-harm (*N* = 5726)

		Highly lethal self-harm^a^	Less lethal self-harm with visible tissue damage^b^	Self-harm without visible tissue damage^c^	Self-harmful behaviors with latency damage^d^	Psychological self-harm^e^
Childhood physical peer victimization	Yes: No	1.48 (1.11, 1.97)**	1.52 (1.28, 1.82)***	1.27 (1.08, 1.48)**	1.27 (1.06, 1.51)**	1.23 (1.03, 1.46)*
Childhood verbal peer victimization	Yes: No	1.11 (0.85, 1.44)	1.25 (1.07, 1.45)**	1.42 (1.24, 1.61)***	1.15 (0.99, 1.34)	1.52 (1.31, 1.76)***
Childhood relational peer victimization	Yes: No	1.17 (0.86, 1.58)	1.69 (1.42, 2.00)***	1.87 (1.61, 2.18)***	2.24 (1.89, 2.65)***	1.90 (1.61, 2.25)***
Physical abuse	Yes: No	0.98 (0.66, 1.45)	0.96 (0.73, 1.26)	1.24 (0.97, 1.59)	1.09 (0.83, 1.42)	1.45 (1.12, 1.88)**
Emotional abuse	Yes: No	1.58 (1.09, 2.30)*	1.14 (0.90, 1.46)	1.06 (0.84, 1.33)	0.90 (0.70, 1.15)	1.15 (0.90, 1.46)
Sexual abuse	Yes: No	1.76 (1.27, 2.45)***	1.67 (1.33, 2.10)***	1.33 (1.07, 1.65)*	1.61 (1.28, 2.03)***	1.30 (1.03, 1.63)*
Physical neglect	Yes: No	1.43 (0.97, 2.11)	1.30 (0.98, 1.71)	1.32 (1.01, 1.72)*	1.40 (1.07, 1.85)*	1.39 (1.06, 1.83)*
Emotional neglect	Yes: No	1.79 (1.16, 2.76)**	1.17 (0.82, 1.65)	1.07 (0.76, 1.51)	1.15 (0.81, 1.62)	0.92 (0.65, 1.30)
Family life stress event scores	Ref: 0					
	1–2	0.99 (0.75, 1.32)	1.63 (1.37, 1.95)***	1.67 (1.44, 1.93)***	1.45 (1.22, 1.73)***	1.85 (1.55, 2.20)***
	3–10	0.84 (0.60, 1.16)	1.97 (1.61, 2.41)***	2.02 (1.70, 2.41)***	1.72 (1.40, 2.10)***	2.13 (1.75, 2.59)***

Note: ^a^adjusted for gender, age, relationship with mother, relationship with father, only child and depression scale scores; ^b^adjusted for gender, age, relationship with mother, relationship with father, only child and depression scale scores; ^c^adjusted for gender, age, self-perceived family status, relationship with mother, relationship with father, only child and depression scale scores; ^d^adjusted for age, self-perceived family status, relationship with mother, relationship with father, only child and depression scale scores; ^e^adjusted for gender, age, self-perceived family status, relationship with mother, relationship with father, only child and depression scale scores

* *P* < 0.05; ** *P* < 0.01; *** *P* < 0.001

## Discussion

### Prevalence of each type of SH

In this study, SH was considered as a series of self-inflicted and intentional behaviours that caused physical and psychological harm. The prevalence rates of highly lethal self-harm, less lethal self-harm with visible tissue damage, self-harm without visible tissue damage, self-harmful behaviours with latency damage and psychological self-harm were 6.1, 20.4, 32.0, 20.0 and 23.0%, respectively. The prevalence of highly lethal self-harm was 6.1% lower than the prevalence of the other four subtypes of SH, which was consistent with previous reports of the prevalence of suicidal attempts ranging from 4.0 to 7.0% in the general adolescent population [33, 38–40]. Moreover, non-suicidal SH (i.e., less lethal self-harm with visible tissue damage and self-harm without visible tissue damage) was consistent with the results of recent studies in China [8, 41] and was higher than the pooled NSSI prevalence of adolescents worldwide [4]. Additionally, our study examined the prevalence rates of self-harmful behaviours with latency damage and psychological self-harm, which accounted for approximately one-fifth each. However, due to the lack of a similar analysis, we could not compare our results with the results of other studies. Although these two SH subtypes may exert less harm on adolescents, future research is required to provide an understanding of the negative impact on adolescent health.

### Childhood maltreatment and SH

All types of childhood maltreatment were linked to SH in adolescents in the univariate analysis. After rigorously adjusting a series of covariates, childhood sexual abuse was still significantly associated with each SH subtype. This finding is consistent with previous studies suggesting that a history of childhood sexual abuse significantly increased the risk of onset and persistence of suicide attempts in adolescents [7, 13]. Additionally, these findings serve as a reminder that childhood sexual abuse may have a more robust relationship with SH than the other risk factors mentioned in our study. Based on this finding, we can deduce that strategies towards the prevention of SH in adolescents need to pay more attention to individuals who have experienced maltreatment during childhood, especially childhood sexual abuse. Additionally, Weierch [42] supported a theoretical model in which posttraumatic stress disorder (PTSD) independently mediated the relationship between childhood sexual abuse and the frequency of NSSI. In this study, we controlled for depression as a covariate. Therefore, future research should focus on other mediators of those relationships to obtain a better understanding of the results in our study and provide suggestions for the prevention of SH.

### Childhood peer victimization and SH

Childhood peer victimization was associated with SH, particularly childhood physical peer victimization, which was consistent with the findings of a meta-analysis that showed positive links between peer victimization and NSSI [17]. Previous studies indicated that younger children who suffered from victimization had more reported NSSI behaviours as children and adolescents [17]. Adolescents who were bullied between seven and 10 years of age had an increased risk for SH in late adolescence, which indirectly led to depression in early adolescence. Moreover, the association between being bullied and SH in adolescents was partially mediated by depression symptoms [9]. However, after adjusting for the depression scale scores in our study, an association still existed between each SH subtype and childhood physical peer victimization. Garisch’s [43] study demonstrated that alexithymia moderated and partially mediated the association between a history of bullying and DSH. In the future, studies are needed to explore the mechanisms underlying the relationship between childhood peer victimization and SH, including mediators and moderators.

### Childhood family life stressful events and SH

An increasing amount of evidence has shown that family life stress elevates the risk for SH. Some research has suggested that stressful life events, such as a single-parent family, parental death or divorce, are important risk factors for SH in adolescents [13, 44]. Studies have also reported that adolescents from families with conflicts, non-intact families, and families with financial hardships were more likely to engage in suicide [18, 19, 45]. Our results indicated that family life stress events were significantly associated with SH but not with highly lethal self-harm in Chinese adolescents. Contradictory findings have reported that the number of negative life events in the previous year increased not only the risk of NSSI [46] but also suicide attempts [47]. Indeed, negative life events experiences may influence the stress system by altering stress systems, such as influencing individuals’ hormones and neurotransmitters, and the subsequent imbalance may lead to suicidal behaviour [48].

### The pattern of the association between childhood adversity and each SH subtype

The association between childhood adversity with highly lethal self-harm identified in our study was different from the associations with the non-lethal SH subtypes. Our study indicated that childhood emotional abuse and neglect were associated with highly lethal self-harm but had no association with the other four SH subtypes. Previous studies found that childhood emotional abuse and neglect were associated with suicidal ideation and attempts in adolescents, particularly for serious suicide attempts [49]. Furthermore, childhood emotional abuse had indirect harmful effects on suicidal behaviours in adolescents [14]. Conversely, regarding the childhood peer victimization and childhood family life stress event scores, highly lethal self-harm was only associated with childhood physical peer victimization, whereas the other four SH subtypes were associated with the three types of childhood victimization and childhood family life stress event scores after the adjustment of covariates. These findings revealed that highly lethal self-harm differs from the other four SH subtypes in its nature. That is likely a consequence of individual genetics over the lifetime, exposure to environmental factors, and the interaction of those two factors. A growing body of studies has reported that suicidal behaviours are associated with a number of genes, including 5-HTTLPR polymorphisms [50], serotonin receptors and transporters, and brain-derived neurotrophic factors (BDNFs) [51], but these findings are controversial. Interactions between genetics and the environment may play significant roles in the risk for suicidal behaviours. Only those environmental factors that are serious or persistent, such as childhood physical peer victimization and sexual abuse, may be regarded as environmental harbouring stressors that affect suicidal behaviours. However, these findings are best regarded as preliminary data that need to be validated in future studies.

### Limitations

The prevalence of a series of self-inflicted and intentional behaviours was explored in this cross-sectional study. We acquired a large and diverse sample. However, the limitations of this study must also be noted to better understand these results. First, this study was a cross-sectional study; therefore, a causal conclusion cannot be drawn. Future studies should use a prospective design to validate these specific relationships. Second, recall bias might affect the accuracy of the results due to the childhood adversity that was experienced in primary school or during an earlier period. Third, the participants in this study were mainly recruited from schools. The few students who dropped out or skipped school missed the investigation. However, because these students might have more health problems and higher risks of SH, future studies should include adolescents from multiple sources. Finally, correlations were found among multiple types of childhood adversity and SH. In this study, we examined only the association between each type of childhood adversity and each type of SH. We did not examine the interaction between several forms of childhood adversity and SH. Future studies could use a more effective method to explore these correlations.

## Conclusions

This study investigated the prevalence of each type of SH among Chinese adolescents by utilizing a large-scale survey sample. The study also examined the association between childhood adversity and SH. We found a high prevalence of SH among Chinese adolescents, which indicated a significant relationship between childhood adversity and SH. Therefore, strategies to prevent SH among adolescents may be more beneficial if they address childhood adversity experiences. Further investigations are needed to explore the mechanisms underlying the association between childhood adversity and different SH subtypes.

## Additional files

Additional file 1: Table S1. Frequency of overlap between different types of self-harm. (DOC 31 kb)

Additional file 2: Table S2. Prevalence of involved in different numbers of self-harm by sample characteristics (*N* = 5726). (DOC 106 kb)

Additional file 3: Table S3. Multivariable logistic regression analysis showing the AOR (95% CI) between childhood adversity and five subtypes of self-harm (*N* = 5726). Results of multivariate logistic regression analysis to confirm the relationships between childhood adversity and each SH subtype, with adjustments for all sociodemographic variables and depression. (DOC 57 kb)

## Abbreviations

AORs: Adjusted odds ratios
BDNFs: Brain-derived Neurotrophic Factors
CI: Confidence intervals
CTQ-SF: Childhood Trauma Questionnaire Short Form
DSH: Deliberate self-harm
DSM-5: Diagnostic and Statistical Manual of Mental Disorders, 5th Edition
EA: Emotional abuse
EN: Emotional neglect
ICD-10: International Classification of Diseases 10th Revision
NSSI: Non-suicidal self-injury
PA: Physical abuse
PN: Physical neglect
PTSD: Posttraumatic stress disorder
SA: Sexual abuse
SDS: Self-Rating Depression Scale
SH: Self-harm
