# Supplementary material for: A self-harm series and its relationship with childhood adversity among adolescents in mainland China: a cross-sectional study
Source: BMC Psychiatry. 2018 Feb 1;18:28. doi: 10.1186/s12888-018-1607-0 (PMC5796511; doi:10.1186/s12888-018-1607-0)
Supplement: Supplementary file 1 — Frequency of overlap between different types of self-harm. (DOC 31 kb) [file 12888_2018_1607_MOESM1_ESM.doc]

**Additional file 1**

**Table S1 Frequency of overlap between different types of self-harm**

| Type of SH | Highly lethal self-harm | Less lethal self-harm with visible tissue damage | Self-harm without visible tissue damage | Self-harmful behaviors with latency damage | Psychological self-harm |
| --- | --- | --- | --- | --- | --- |
|  | n (%) | n (%) | n (%) | n (%) | n (%) |
| Highly lethal self-harm | 349 (100) | 280 (24.0) | 271 (14.8) | 228 (19.9) | 220 (16.7) |
| Less lethal self-harm with visible tissue damage | 280 (80.2) | 1168 (100) | 898 (49.0) | 568 (49.6) | 636 (48.3) |
| Self-harm without visible tissue damage | 271 (77.7) | 898 (76.9) | 1834 (100) | 795 (69.4) | 937 (71.2) |
| Self-harmful behaviors with latency damage | 228 (65.3) | 568 (48.6) | 795 (43.3) | 1145 (100) | 709 (53.9) |
| Psychological self-harm | 220 (63.0) | 636 (54.5) | 937 (51.1) | 709 (61.9) | 1316 (100) |
