# Supplementary material for: A self-harm series and its relationship with childhood adversity among adolescents in mainland China: a cross-sectional study
Source: BMC Psychiatry. 2018 Feb 1;18:28. doi: 10.1186/s12888-018-1607-0 (PMC5796511; doi:10.1186/s12888-018-1607-0)
Supplement: Supplementary file 2 — Prevalence of involved in different numbers of self-harm by sample characteristics (N = 5726). (DOC 106 kb) [file 12888_2018_1607_MOESM2_ESM.doc]

**Additional file 2**

**Table S2** Prevalence of involved in different numbers of self-harm by sample characteristics (*N* = 5726)

| Variables | Non-involved in self-harm | One type of self-harm | Two types of self-harm | Three types of self-harm | Four or five types of self-harm | *χ^2^* value | *P* value |
| --- | --- | --- | --- | --- | --- | --- | --- |
|  | n (%) | n (%) | n (%) | n (%) | n (%) |  |  |
| Gender | | | | | | 54.2 | <0.001 |
| Female | 1467 (51.0) | 449 (15.6) | 412 (14.3) | 278 (9.7) | 272 (9.5) |  |  |
| Male | 1664 (58.4) | 478 (16.8) | 321 (11.3) | 205 (7.2) | 180 (6.3) |  |  |
| Age |  |  |  |  |  | 123.3 | <0.001 |
| 12 years | 568 (64.8) | 142 (16.2) | 91 (10.4) | 45 (5.1) | 30 (3.4) |  |  |
| 13 years | 493 (53.6) | 162 (17.6) | 118 (12.8) | 87 (9.5) | 59 (6.4) |  |  |
| 14 years | 489 (58.1) | 125 (14.8) | 102 (12.1) | 58 (6.9) | 68 (8.1) |  |  |
| 15 years | 464 (57.8) | 101 (12.6) | 96 (12.0) | 71 (8.8) | 71 (8.8) |  |  |
| 16 years | 469 (52.6) | 152 (17.0) | 114 (12.8) | 78 (8.7) | 79 (8.9) |  |  |
| 17 years | 471 (47.3) | 162 (16.3) | 157 (15.8) | 102 (10.2) | 104 (10.4) |  |  |
| 18 years | 177 (44.5) | 83 (20.9) | 55 (13.8) | 42 (10.6) | 41 (10.3) |  |  |
| Self-perceived family status | | | | | | 19.9 | 0.011 |
| Bad | 397 (49.6) | 121 (15.1) | 117 (14.6) | 87 (10.9) | 78 (9.8) |  |  |
| General | 2427 (55.4) | 712 (16.3) | 557 (12.7) | 350 (8.0) | 331 (7.6) |  |  |
| Good | 307 (55.9) | 94 (17.1) | 59 (10.7) | 46 (8.4) | 43 (7.8) |  |  |
| Relationship with mother | | | | | | 43.7 | <0.001 |
| Good | 2476 (56.6) | 700 (16.0) | 552 (12.6) | 342 (7.8) | 303 (6.9) |  |  |
| Poor | 655 (48.4) | 227 (16.8) | 181 (13.4) | 141 (10.4) | 149 (11.0) |  |  |
| Relationship with father | | | | | | 77.1 | <0.001 |
| Good | 2226 (58.3) | 612 (16.0) | 426 (11.2) | 285 (7.5) | 266 (7.0) |  |  |
| Poor | 905 (47.4) | 315 (16.5) | 307 (16.1) | 198 (10.4) | 186 (9.7) |  |  |
| Only child | | | | | | 55.8 | <0.001 |
| Yes | 1473 (59.7) | 386 (15.6) | 275 (11.1) | 191 (7.7) | 144 (5.8) |  |  |
| No | 1658 (50.9) | 541 (16.6) | 458 (14.1) | 292 (9.0) | 308 (9.5) |  |  |
| Family structure | | | | | | 16.5 | 0.170 |
| Nuclear family | 2096 (55.3) | 607 (16.0) | 480 (12.7) | 302 (8.0) | 307 (8.1) |  |  |
| Lager family | 724 (53.5) | 239 (17.7) | 172 (12.7) | 117 (8.6) | 101 (7.5) |  |  |
| Single-parent family | 253 (53.9) | 65 (13.9) | 70 (14.9) | 50 (10.7) | 31 (6.6) |  |  |
| Other | 58 (51.8) | 16 (14.3) | 11 (9.8) | 14 (12.5) | 13 (11.6) |  |  |
| Childhood physical peer victimization | | | | | | 244.4 | <0.001 |
| Yes | 480 (39.0) | 216 (17.5) | 173 (14.0) | 175 (14.2) | 188 (15.3) |  |  |
| No | 2651 (59.0) | 711 (15.8) | 560 (12.5) | 308 (6.9) | 264 (5.9) |  |  |
| Childhood verbal peer victimization | | | | | | 275.4 | <0.001 |
| Yes | 1017 (42.4) | 471 (19.6) | 359 (15.0) | 281 (11.7) | 273 (11.4) |  |  |
| No | 2114 (63.6) | 456 (13.7) | 374 (11.2) | 202 (6.1) | 179 (5.4) |  |  |
| Childhood relational peer victimization | | | | | | 588.4 | <0.001 |
| Yes | 507 (32.0) | 299 (18.9) | 271 (17.1) | 236 (14.9) | 270 (17.1) |  |  |
| No | 2624 (63.3) | 628 (15.2) | 462 (11.2) | 247 (6.0) | 182 (4.4) |  |  |
| Physical abuse | | | | | | 219.5 | <0.001 |
| Yes | 125 (29.4) | 75 (17.6) | 59 (13.9) | 68 (16.0) | 98 (23.1) |  |  |
| No | 3006 (56.7) | 852 (16.1) | 674 (12.7) | 415 (7.8) | 354 (6.7) |  |  |
| Emotional abuse | | | | | | 407.0 | <0.001 |
| Yes | 178 (28.2) | 98 (15.5) | 95 (15.0) | 104 (16.5) | 157 (24.8) |  |  |
| No | 2953 (58.0) | 829 (16.3) | 638 (12.5) | 379 (7.4) | 295 (5.8) |  |  |
| Sexual abuse | | | | | | 297.2 | <0.001 |
| Yes | 161 (32.3) | 70 (14.0) | 68 (13.6) | 71 (14.2) | 129 (25.9) |  |  |
| No | 2970 (56.8) | 857 (16.4) | 665 (12.7) | 412 (7.9) | 323 (6.2) |  |  |
| Physical neglect | | | | | | 353.7 | <0.001 |
| Yes | 98 (25.9) | 60 (15.8) | 43 (11.3) | 63 (16.6) | 115 (30.3) |  |  |
| No | 3033 (56.7) | 867 (16.2) | 690 (12.9) | 420 (7.9) | 337 (6.3) |  |  |
| Emotional neglect | | | | | | 275.9 | <0.001 |
| Yes | 54 (23.7) | 29 (12.7) | 29 (12.7) | 37 (16.2) | 79 (34.6) |  |  |
| No | 3077 (56.0) | 898 (16.3) | 704 (12.8) | 446 (8.1) | 373 (6.8) |  |  |
| Family life stress event scores | | | | | | 415.9 | <0.001 |
| 0 | 1444 (70.5) | 251 (12.2) | 174 (8.5) | 92 (4.5) | 88 (4.3) |  |  |
| 1-2 | 1186 (50.7) | 424 (18.1) | 325 (13.9) | 217 (9.3) | 185 (7.9) |  |  |
| 3-10 | 501 (37.4) | 252 (18.8) | 234 (17.5) | 174 (13.0) | 179 (13.4) |  |  |
| Depression | | | | | | 189.4 | <0.001 |
| Yes | 1852 (49.9) | 582 (15.7) | 501 (13.5) | 386 (10.4) | 390 (10.5) |  |  |
| No | 1279 (63.5) | 345 (17.1) | 232 (11.5) | 97 (4.8) | 62 (3.1) |  |  |

Note: Four types of self-harm and five types of self-harm were merged due to small sample so as to enlarge the power of Chi-square test.
