# Supplementary material for: A self-harm series and its relationship with childhood adversity among adolescents in mainland China: a cross-sectional study
Source: BMC Psychiatry. 2018 Feb 1;18:28. doi: 10.1186/s12888-018-1607-0 (PMC5796511; doi:10.1186/s12888-018-1607-0)
Supplement: Supplementary file 3 — Multivariable logistic regression analysis showing the AOR (95% CI) between childhood adversity and five subtypes of self-harm (N = 5726). Results of multivariate logistic regression analysis to confirm the relationships between childhood adversity and each SH subtype, with adjustments for all sociodemographic variables and depression. (DOC 57 kb) [file 12888_2018_1607_MOESM3_ESM.doc]

**Additional file 3**

**Table S3** Multivariable logistic regression analysis showing the AOR (95% CI) between childhood adversity and five subtypes of self-harm (*N* = 5726)

|  | | Highly lethal self-harm | Less lethal self-harm with visible tissue damage | | Self-harm without visible tissue damage | Self-harmful behaviors with latency damage | Psychological self-harm |
| --- | --- | --- | --- | --- | --- | --- | --- |
| Childhood physical peer victimization | Yes: No | 1.48 (1.11, 1.98)** | | 1.52 (1.28, 1.82)*** | 1.27 (1.08, 1.48)** | 1.28 (1.07, 1.53)** | 1.23 (1.03, 1.46)* |
| Childhood verbal peer victimization | Yes: No | 1.11 (0.86, 1.45) | | 1.25 (1.07, 1.46)** | 1.41 (1.24, 1.61)*** | 1.15 (0.99, 1.35) | 1.51 (1.31, 1.76)*** |
| Childhood relational peer victimization | Yes: No | 1.15 (0.85, 1.57) | | 1.68 (1.42, 2.00)*** | 1.87 (1.61, 2.18)*** | 2.23 (1.88, 2.65)*** | 1.90 (1.61, 2.25)*** |
| Physical abuse | Yes: No | 0.98 (0.66, 1.47) | | 0.96 (0.73, 1.27) | 1.25 (0.97, 1.60) | 1.09 (0.84, 1.43) | 1.45 (1.12, 1.88)** |
| Emotional abuse | Yes: No | 1.58 (1.09, 2.30)* | | 1.14 (0.89, 1.45) | 1.06 (0.84, 1.33) | 0.89 (0.69, 1.14) | 1.15 (0.90, 1.46) |
| Sexual abuse | Yes: No | 1.73 (1.24, 2.41)** | | 1.64 (1.31, 2.07)*** | 1.33 (1.07, 1.65)* | 1.62 (1.28, 2.03)*** | 1.30 (1.03, 1.63)* |
| Physical neglect | Yes: No | 1.43 (0.97, 2.10) | | 1.29 (0.97, 1.70) | 1.32 (1.01, 1.71)* | 1.40 (1.07, 1.84)* | 1.39 (1.06, 1.82)* |
| Emotional neglect | Yes: No | 1.83 (1.19, 2.83)** | | 1.18 (0.83, 1.67) | 1.06 (0.75, 1.50) | 1.15 (0.81, 1.62) | 0.92 (0.65, 1.30) |
| Family life stress event scores | Ref: 0 | | | | | | |
|  | 1-2 | 1.01 (0.76, 1.34) | | 1.67 (1.40, 2.00)*** | 1.67 (1.44, 1.94)*** | 1.45 (1.21 1.73)*** | 1.85 (1.55, 2.20)*** |
|  | 3-10 | 0.86 (0.61, 1.21) | | 2.06 (1.68, 2.53)*** | 2.03 (1.70, 2.41)*** | 1.72 (1.40, 2.11)*** | 2.14 (1.75, 2.61)*** |

Note: adjusted for gender, age, self-perceived family status, relationship with mother, relationship with father, family structure, only child and depression scale scores.

* *P* < 0.05; ** *P* < 0.01; *** *P* < 0.001.
